# Immobilization of Low-Cost Alternative Vegetable Peroxidase (*Raphanus sativus* L. peroxidase): Choice of Support/Technique and Characterization

**DOI:** 10.3390/molecules25163668

**Published:** 2020-08-12

**Authors:** Gabrielle Souza da Silva Barbosa, Maria Emanuela P. S. Oliveira, Ana Beatriz S. dos Santos, Osmar Calderón Sánchez, Cleide Mara Faria Soares, Alini Tinoco Fricks

**Affiliations:** 1 Programa de Pós-Graduação em Biotecnologia Industrial, Tiradentes University, 49032-490 Aracaju, SE, Brazil; gabriellesosi@hotmail.com (G.S.d.S.B.); emanuelabiomed@hotmail.com (M.E.P.S.O.); anabeatrizsantos351@gmail.com (A.B.S.d.S.); cleide18@yahoo.com.br (C.M.F.S.); 2 Laboratory of Bioprocess Engineering, Institute of Technology and Research, Farolândia, 49032-490 Aracaju, SE, Brazil; 3 Laboratory of Organic Synthesis, Faculty of Chemistry, La Habana University, 10400 La Habana, Cuba; calderonsanchez@yahoo.com

**Keywords:** radish peroxidase, immobilization, supports, characterization

## Abstract

In the present work the radish (*Raphanus sativus* L.) was used as the low-cost alternative source of peroxidase. The enzyme was immobilized in different supports: coconut fiber (CF), calcium alginate microspheres (CAMs) and silica SBA-15/albumin hybrid (HB). Physical adsorption (PA) and covalent binding (CB) as immobilization techniques were evaluated. Immobilized biocatalysts (IBs) obtained were physicochemical and morphologically characterized by SEM, FTIR and TGA. Also, optimum pH/temperature and operational stability were determined. For all supports, the immobilization by covalent binding provided the higher immobilization efficiencies—immobilization yield (IY%) of 89.99 ± 0.38% and 77.74 ± 0.42% for HB and CF, respectively. For CAMs the activity recovery (AR) was of 11.83 ± 0.68%. All IBs showed optimum pH at 6.0. Regarding optimum temperature of the biocatalysts, HB-CB and CAM-CB maintained the original optimum temperature of the free enzyme (40 °C). HB-CB showed higher operational stability, maintaining around 65% of the initial activity after four consecutive cycles. SEM, FTIR and TGA results suggest the enzyme presence on the IBs. Radish peroxidase immobilized on HB support by covalent binding is promising in future biotechnological applications.

## 1. Introduction

Peroxidases (EC 1.11.1.7) are enzymes that catalyze the oxidation of a wide variety of substrates dependent on H_2_O_2_ [1]. Among vegetable peroxidases, horseradish peroxidase (HRP) is the most studied [2,3] due to the numerous possibilities of applications, such as bioremediation, biosensors and diagnostic kits [4,5]. In addition, HRP presents considerable stability [6]. However, its high commercial cost can inhibit the use on an industrial scale [7]. One strategy to break this barrier is to look for alternative plant peroxidases—similar to HRP—that can perform the same applications. Thus, many studies focused on applications for alternative peroxidases have been developed: peroxidase from guinea grass leaves as biosensor [8], turnip peroxidase for analytical and diagnostic kits [9], radish and turnip peroxidases in organic synthesis [10], cedar leaf peroxidase for decolorization of dyes [11], and potato pulp and mesquite (*Prosopis juliflora*) peroxidases for bioremediation of phenolic compounds [12,13]. 

Just like horseradish, the radish (*Raphanus sativus* L.) is classified as belonging to the Brassicaceae family [14]. Radish peroxidase presents 70% of similarity in respect to primary sequence amino acids of the HRP [7]. Also, radish is one of the vegetables most commonly cultivated around the world, especially in Eastern Asia [15,16], and presents convenient agronomic characteristics, such as: the high resistance to climatic conditions, short time and little area necessaries for planting [7]. Thus, the radish consists a promising alternative source of low-cost plant peroxidase. In this way, several studies have been development with crude peroxidase from radish [7,17,18,19]. 

Among the main advantages of the use of peroxidases in industrial process is the ecologic approach that contributes to the development of environmentally sustainable processes [20] and high specificity and high selectivity for substrates [21]. However, the industrial applications of the enzymes in free form are limited due to the impossibility of reuse and instability, as for example, at higher temperatures and pHs that are not equal to the physiological environment, low stability in organic solvents and storage [21,22,23,24]. Enzyme immobilization can provide stability to the biocatalyst. One way to achieve this goal is to use a support with favorable physical and chemical characteristics (active groups for enzyme interaction, pore size, specific area and internal morphology) in order to intensity the protein/support interactions. A successful enzyme immobilization process involves maintaining or conformational change of the enzyme structure to a more active form. Thus, the catalytic activity should be maintained or increase [25,26]. Also, as result of the enzyme stabilization, the immobilized enzyme can tune the selectivity and specificity of the biocatalyst; can minimize problems regarding to enzyme inhibition that can occur at high substrate concentration or co-products presence [27,28,29]. The choice of a support for enzyme immobilization must also take into account aspects related to physical-chemical stability, sustainability and cost-effective [30,31,32,33].

Different techniques can be used for enzymes immobilization. Physical adsorption and covalent binding are consolidated techniques commonly used for immobilization of enzymes on supports [34]. The literature reports the immobilization of commercial peroxidase (HRP) by covalent binding (CB) and physical adsorption (PA) in a lignocellulosic material—sugarcane bagasse—as organic support [35]. In the same way, other enzymes were immobilized on coconut fiber [36,37,38,39], an important bio-residue of world agribusiness [40,41]. Calcium alginate microspheres also were used as organic support for enzyme immobilization due to be material of easy synthesis, inexpensive and non-toxic [42,43,44]. Inorganic supports of distinct chemical natures have been applied for enzymes immobilization, such as iron hydroxyl oxide [45] and silica [31]. Among inorganic supports the materials silica-based are the most usual choices [31] due to large pore volume, high surface area, high thermal stability and mechanical resistance [28,46]. The literature reports the use of silica SBA-15 and SBA-16 as support for immobilization of HRP [47,48,49]. 

In addition to organic and inorganic supports, the so-called hybrid organic-inorganic supports (HB) have been used successfully in the immobilization of enzymes [50]. HB supports combine organic and inorganic components during its synthesis that allows to form new materials with improved properties and minimizes component individual limitations [51].

Thus, the present work reports the immobilization of crude radish peroxidase—a low-cost enzyme—by physical adsorption (PA) and covalent binding (CB) techniques on different supports: coconut fiber (CF), calcium alginate microspheres (CAMs) and hybrid SBA-15/albumin (HB). Considering the potential for further applications, biochemical parameters and physicochemical and morphological characterization were also studied. 

## 2. Results and Discussion

The results report the immobilization of radish (*Raphanus sativus* L.) peroxidase in the coconut fiber (CF) and calcium alginate microspheres (CAMs) supports by physical adsorption (PA) and covalent binding (CB) techniques and in SBA-15/albumin hybrid (HB) support by the covalent binding (CB) technique. 

### 2.1. Effect of Protein Loading

**Figure 1:**
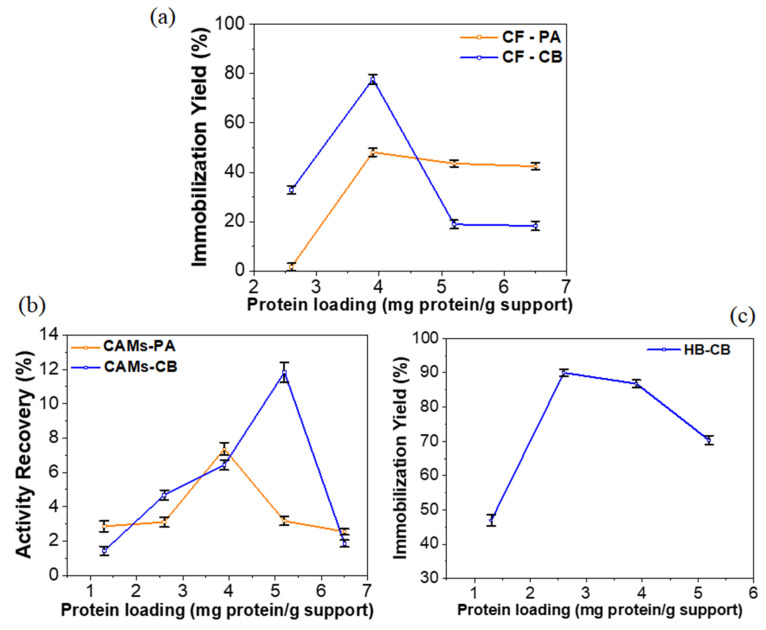
Effect of protein loading (1.2 to 6.5 mg protein/g support) on the radish peroxidase immobilization by physical Adsorption (PA) and covalent binding (CB) techniques.: (**a**) Coconut fiber (CF), (**b**) calcium alginate microspheres (CAMs), (**c**) SBA-15/albumin hybrid (HB).

The effect of protein loading on a support for the purpose of enzyme immobilization should be evaluated, especially when different supports and techniques are investigated. Protein loading effect was evaluated in a range of 1.3 to 6.5 mg protein/g support, as shown the Figure 1. All immobilization efficiencies of the IBs were statistically significant (*p* < 0.05). For all supports and immobilization techniques investigated the increase in protein load resulted in an increase of the immobilization efficiency, reaching a highest value followed by a decrease in this value. These results indicate the saturation of the supports due to enzyme overload. Also, an exaggerated protein load—that include interest enzyme for immobilization and other proteins molecules—can block the access of the substrate to the active site of the enzyme and reduce activity of the immobilized system [26].

The immobilized biocatalysts (IBs) CF-CB, CF-PA and CAM-PA reached the higher immobilization yields (IY) 77.74 ± 0.42%, 48.15 ± 0.36% and activity recovery (AR) of 7.36 ± 0.37%, respectively, at protein loading of 3.9 mg protein/g support (Figure 1a,b). For CAM-CB the maximum AR was only 11.83 ± 0.68% even at high protein loading (5.2 mg protein/g support) (Figure 1b). Crude radish peroxidase was immobilized with high IY (89.99 ± 0.38%) on SBA-15/albumin hybrid support by covalent binding (HB-CB) with a low protein load (2.6 mg protein/g support). In contrast to obtained results with CF and CAM, even with an increased protein load (5.2 mg protein/g support) a considerable IY was reached (70.30 ± 0.45%) (Figure 1c). The best result of IY for HB-CB obtained with the low protein load shows that this condition favors peroxidase-support interaction on the immobilization process. 

Appropriate immobilization technique can optimize the catalytic performance of immobilized enzymes. The best results of immobilization the crude radish peroxidase on CF and CAMs supports were reached by covalent binding (CB) technique compared to physical adsorption (PA). Immobilization by CB provides strong enzyme-support binding that prevents enzyme leaching and attenuates the loss of enzyme active sites [52].

The result of immobilization of crude radish peroxidase on the CF support by CB technique was promising (IY: 77.74 ± 0.42%), compared to the literature. Commercial HRP was immobilized on sugarcane bagasse by CB technique obtaining 35% of IY [35]. Bezerra et al. [36] immobilized a fungal laccase on coconut fiber obtaining IY of 98%. 

**Figure 2:**
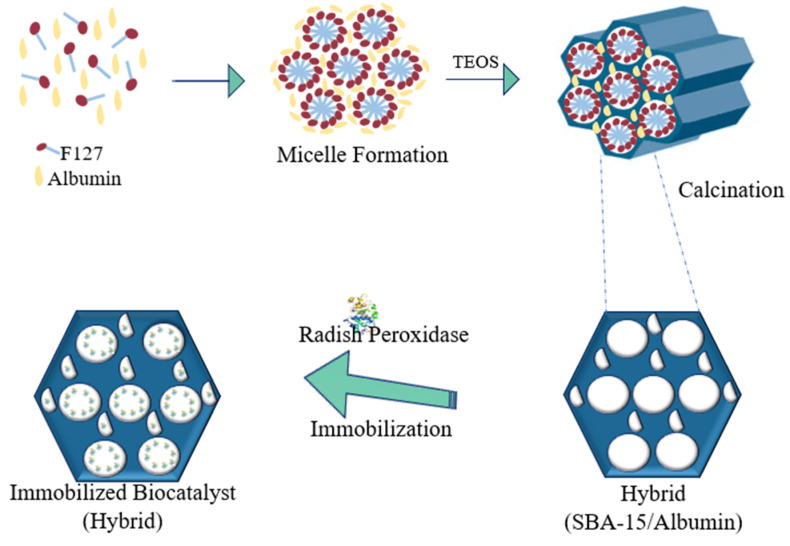
Proposed scheme of synthesis of hybrid (SBA-15/albumin) and immobilization of the enzyme.

Among investigated supports, the SBA-15/albumin hybrid (HB) showed be the most appropriate for immobilization of crude radish peroxidase (IY: 89.99 ± 0.38%). Inorganic support SBA-15 is mesoporous material of large hexagonal and ordered pores [47]. HB synthesis conforms proposed methodology consists in a support even more porous than the conventional SBA-15. This is because in addition to structure-directing agent being calcined, the albumin added during the synthesis of the support is also calcined, increasing the porosity of the material. Thus, the surface area of the support acquires a suitable geometry in terms of attachment of the enzyme to the support in the immobilization process [53]. Figure 2 shows the proposed scheme of the synthesis of hybrid (SBA-15/albumin) and immobilization of the enzyme. 

### 2.2. Effect of pH

The catalytic activity of an enzyme is influenced by pH because the enzyme-substrate interaction can be altered [54]. The effect of pH on immobilized enzymes must be evaluated considering the possibility of applying them on an industrial scale.

**Figure 3:**
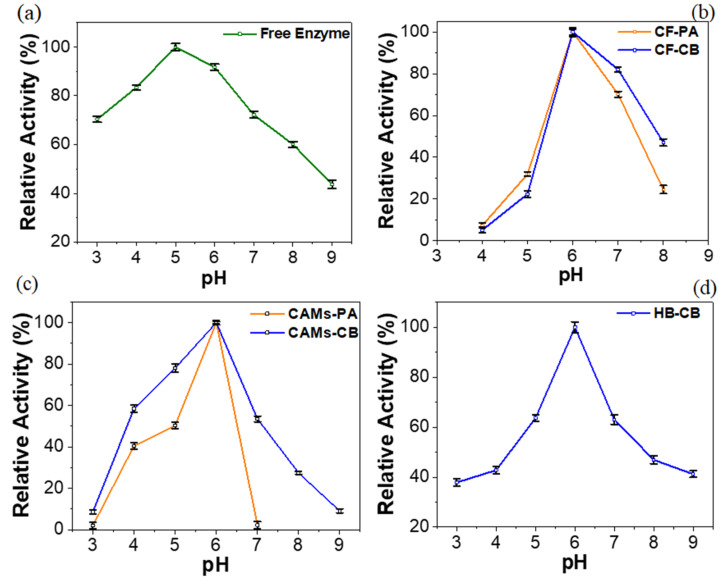
Effect of pH (3.0–9.0) on the radish peroxidase activity. (**a**) Free enzyme. Immobilized biocatalysts by physical Adsorption (PA) and covalent Binding (CB) techniques: (**b**) coconut fiber (CF), (**c**) calcium alginate microspheres (CAMs), (**d**) SBA-15/albumin hybrid (HB).

Figure 3 shows the effect of pH (3.0 to 9.0) on the activities of free and immobilized radish peroxidases on different supports. All relative activities (free radish peroxidase and IBs) were statistically significant (*p* < 0.05). Free radish peroxidase showed maximum activity at pH 5.0 (Figure 3a), the same optimal pH reported for HRP [55]. This result denotes the similarity in the biochemical characteristics of these enzymes. Also, according to literature most peroxidases exhibiting optimum activity at acid pH (4.5–6.5) [1]. 

The optimum pH for all IBs was shifted to 6.0 (Figure 3b–d). Considering vegetable peroxidases, this increase on the optimum pH value is recurrent. HRP immobilized in wool showed optimum pH at 7.0 [56]. Radish peroxidase immobilized on CF by CB maintained activity above to 60% at pH 7.0, while the immobilized enzyme on CAMs these values were observed at acid pHs (4.0 and 5.0). HB-CB showed stability at pH between 5–7 with relative activity greater than 60%. The unequal partitioning of H^+^ and OH^−^ concentrations in the microenvironment of the immobilized enzyme, due to electrostatic interactions with the support, can induce changes in the optimum pH [57]. The reduction of the enzymatic activity observed at extreme pHs has possibly occurred by the denaturation of enzymes by ionization [58]. 

### 2.3. Effect of Temperature

**Figure 4:**
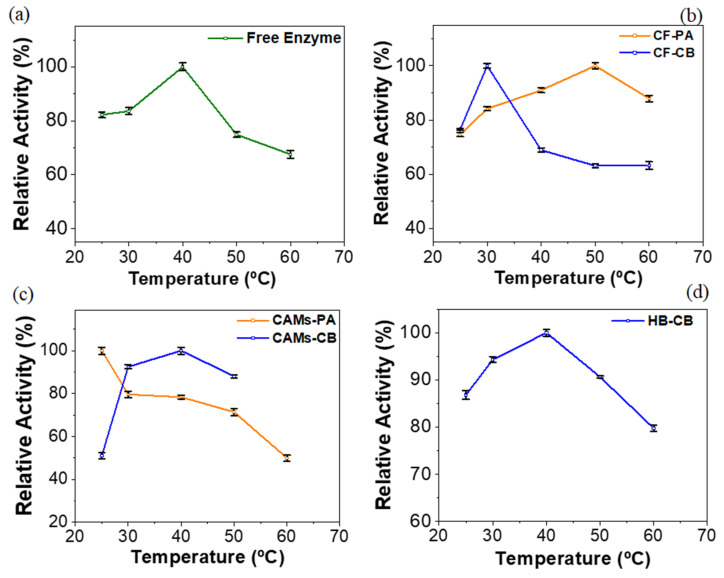
Effect of temperature (25–60 °C) on the radish peroxidase activity. (**a**) Free enzyme. Immobilized biocatalysts by physical Adsorption (PA) and covalent Binding (CB) techniques: (**b**) coconut fiber (CF), (**c**) calcium alginate microspheres (CAMs) and (**d**) SBA-15/albumin hybrid (HB).

The effect of temperature for free enzyme and IBs was evaluated in a range of 25–60 °C. The results are showed in terms of relative activity (RA) compared to the maximum activity (Figure 4). All relative activities (free radish peroxidase and IBs) were statistically significant (*p* < 0.05). Free radish peroxidase showed an increase in enzymatic activity as the temperature increased in the range of 25–40 °C. At 50 and 60 °C the enzyme showed a decrease in enzyme activity (Figure 4a). The optimum temperature for free radish peroxidase (40 °C) was the same reported by the literature for horseradish peroxidase [59].

The immobilization of an enzyme can alter its thermal stability. An immobilized biocatalyst should preferably exhibit high catalytic activity over a wide temperature range; however, this is not always achieved. Immobilized radish peroxidase on CF by PA (IY: 48.15 ± 0.36%) showed optimum activity at 50 °C and maintained RA close to 89.99 ± 0.38% at 40 and 60 °C, while the immobilized enzyme by CB showed optimum temperature at 30 °C. Thus, CF-PA proved to be more resistant at high temperatures—on the range studied—compared the others IBs (Figure 4b). This result seems contradictory has seen that the radish peroxidase immobilization by covalent binding in coconut fiber has resulted in higher IY value (77.74 ± 0.42%). However, with regard to the immobilization of enzymes by covalent binding, it is important to note that the strong bind established between the activated support and the enzyme can cause conformational changes in the biocatalyst that can compromise or improve the thermal stability of the immobilized biocatalyst. Similar result is described in the literature for immobilized enzymes on activated supports with glutaraldehyde [60].

Immobilized radish peroxidase on CAMs by PA showed optimum temperature at 25 °C. From 30 °C the enzyme gradually loses activity. In contrast, CAMs-CB exhibited increase on activity with increased temperature from 30 to 40 °C (maximum activity at 40 °C). At 50 °C was observed a decreasing on activity (Figure 4c). The results are according to literature. Jamal et al. [61] determined the optimum temperature of an immobilized peroxidase in alginate/pectin gel by entrapment. As well in the present study, the enzyme showed higher activity at 40 °C. 

For immobilized biocatalyst HB-CB the temperature influenced the peroxidase activity in similar way to observed on the free radish peroxidase—optimum temperature 40 °C. Also, at 50 and 60 °C the relative activities were greater than 80%, a better result than the free enzyme (Figure 4d). This result suggests that radish peroxidase immobilization on HB by CB improve the thermal stability of biocatalyst, according to observed on literature [49].

### 2.4. Operational Stability

**Figure 5:**
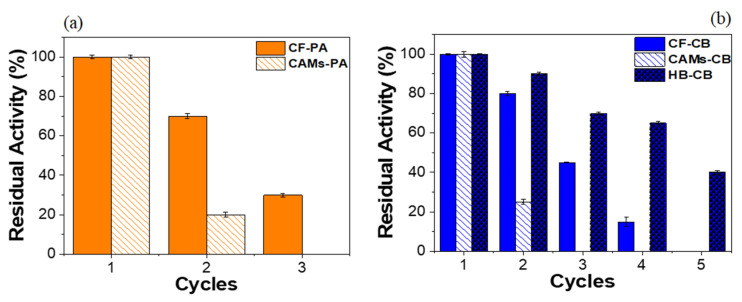
Operational Stability for immobilized radish peroxidase on coconut fiber (CF), calcium alginate microspheres (CAMs) and SBA-15/albumin hybrid (HB): (**a**) physical adsorption (PA), (**b**) covalent binding (CB).

Immobilized enzymes should be evaluated for operational stability in order to verify their application potential. The possibility of reuse minimizes costs in bioprocesses [62] and consolidates the stability of the immobilized enzyme [63]. Operational stability data for IBs are showed in Figure 5. All residual activities of the IBs were statistically significant (*p* < 0.05). As shows Figure 5a, the radish peroxidase immobilized in CF by PA maintained 70.83 ± 0.83% of the initial activity in the second cycle, however on third cycle the residual activity was only 30.70 ± 0.68%. Despite its considerable activity relative at 50 and 60 °C (Figure 4b), CF-PA showed low operational stability that limits future applications. CAMs-PA expressed only 19.99 ± 0.92% of residual activity after second cycle of use. Enzyme immobilization by PA technique involves weak interactions enzyme-support which can cause leaching of the enzyme adsorbed on the support [64] resulting low operational stability.

Figure 5b shows the results of operational stability for radish crude peroxidase immobilized by CB on CF, CAMs and HB supports. Immobilized enzyme in CF retained about 80.27 ± 1.01% of activity after 2 cycles. After the third reuse, the residual activity was 44.94 ± 0.23%. CAMs-CB showed low operational stability: second cycle showed only 24.88 ± 1.24% residual activity. Furthermore, in the third reuse the microspheres showed low mechanical strength, making it difficult to recover from the reaction medium. HB-CB operational stability results showed the highest number of reuse cycles. After 4 reaction cycles the enzyme exhibited 65.36 ± 0.71% of residual activity, with 40.04 ± 0.76% activity in the fifth reuse. These results demonstrate that the higher IY obtained for HB-CB (89.99 ± 0.38) may contribute to the improvement of the operational stability of IB. Also, according Zdarta et al. [31], hybrid support can result in a reusable and stable biocatalytic system. 

Operational stability data corroborated the higher values of efficiencies immobilization for the IBs obtained by CB technique. Usually the reuse capacity of an immobilized enzyme by physical adsorption is smaller than the covalently immobilized. In the CB technique, after the functionalization with APTS, the functional groups of the support are pre-activated in the presence of glutaraldehyde. That way enzyme-support interactions are maximized, therefore minimizing the loss of enzyme molecules from the support with reuse cycles. According to literature the use of glutaraldehyde as a functionalizing agent of supports provides greater operational stability on immobilized biocatalysts [65]. The decreasing residual activity of the IBs in the subsequent cycles of reuse probably occurs due to the denaturation of the enzyme [66]. 

### 2.5. Physicochemical and Morphological Characterization

#### 2.5.1. Scanning Electron Microscopy (SEM)

**Figure 6:**
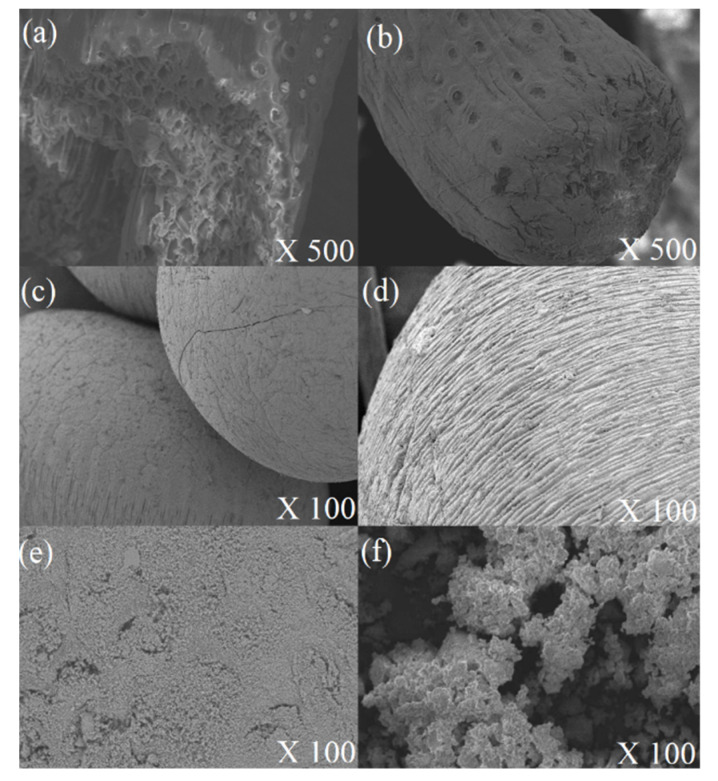
Scanning electron microscopy (SEM) image. Supports/Immobilized biocatalysts by covalent binding (CB): coconut fiber (**a**); coconut fiber-CB (**b**); calcium alginate microspheres (**c**); calcium alginate microspheres-CB (**d**); SBA-15/albumin hybrid (**e**); SBA-15/albumin-CB (**f**).

In order to identify the morphological characteristics of the supports and immobilized radish peroxidase by CB the scanning electron microscopy (SEM) was performed, as shown in the Figure 6. SEM data suggest that the alkaline treatment of the CF was efficient. Image suggests removal of impurities because shows a regular/smooth surface (Figure 6a). According to literature, alkaline treatment of lignocellulosic materials promotes the partial removal of the lignin, accentuating the porosity of the material and expositing the cellulose fibrils. Also, glucan and xylan contents are increased, contributing to greater porosity and the internal area [67]. The morphological characteristics described associates to immobilization by covalent binding may be related to high IY value (77.74 ± 0.42%) obtained on CF-CB, according to literature [36]. CF-CB image shows a covering of the cellulose fibrils (Figure 6b), indicating possible interactions between CF support and free amine of the enzyme.

The formation of calcium alginate microspheres (CAMs) by ionotropic gelation of sodium alginate is established in the literature [68]. Figure 6c shows a relatively smooth and uniform surface for the CAMs support. In contrast, the SEM image of CAMs-CB (Figure 6d) shows a surface with high roughness. This morphological alteration suggests the occurrence of the enzyme-support interaction. However, the possible formation of multi-layers of the protein in the immobilized biocatalyst, as the image suggests, can compromise the access of the substrate to the active site of the enzyme, which contributed to the low value in the recovery of activity (RA: 11.83 ± 0.68%). 

SBA15/albumin (HB) support image is shown in Figure 6e. Synthesis process of the HB support includes calcination and the granulometry control by sieving. In this way, HB has a configuration of powder. HB-CB image shows that radish peroxidase immobilization culminated in agglomerate forming. However, structural configuration of the material was maintained (Figure 6f). Thus, the result suggests enzyme-HB interaction and corroborates with high IY (89.99 ± 0.38%) value obtained on HB-CB. 

#### 2.5.2. Fourier-Transform Infrared Spectroscopy (FTIR)

**Figure 7:**
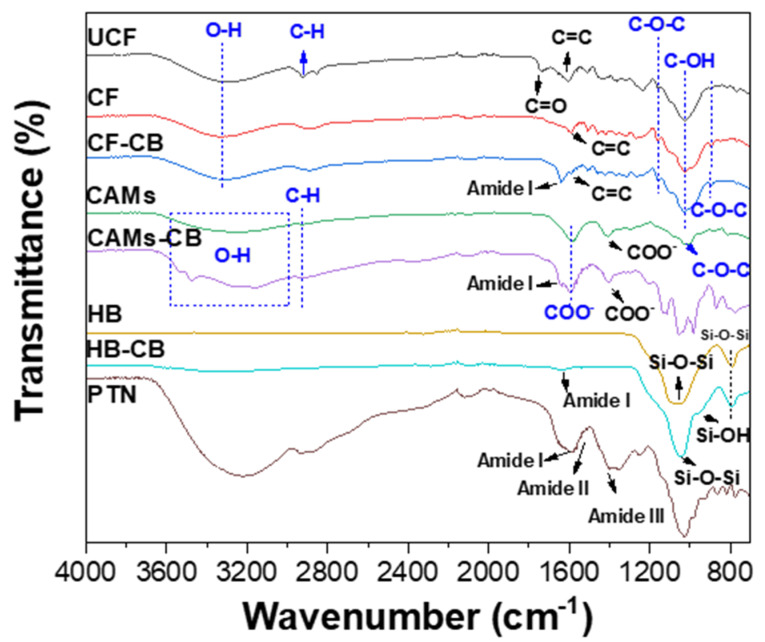
Fourier Transform Infrared Spectroscopy (FTIR) analysis. Supports: untreated coconut fiber (UCF), treated coconut fiber (CF), calcium alginate microspheres (CAMs) and SBA-15/albumin hybrid (HB). Immobilized radish peroxidase by covalent binding (CB): HB-CB, CAMs-CB, CF-CB. Radish protein (PTN).

FTIR spectra of radish protein (PTN), supports (untreated coconut fiber (UCF), treated coconut fiber (CF), calcium alginate microspheres (CAMs), SBA-15/albumin hybrid (HB)) and immobilized radish peroxidase by covalent binding (CB) (HB-CB, CAMs-CB, CF-CB) are shown in Figure 7. 

PTN spectrum showed characteristic absorption bands of the protein: 1655 cm^−1^ (amide I) related to α-helix structure [45]; 1580 cm^−1^ (amide II) corresponding to N-H bending and C-N stretching [69] and 1400 cm^−1^ (amide III) related to several modes of NH [36].

UCF, CF and CF-CB spectra showed typical vibration bands of the lignocellulosic fraction. For all CF spectra an absorption band at 897 cm^−1^ was observed. This band is attributed to the C-O-C stretching referring to glycosidic binds between the sugar units of the cellulosic structure [35]. Also, bands at 1160 cm^−1^, referring to asymmetrical stretching C-O-C [36], and more intense band in 1028 cm^−1^, associated to C-OH stretch vibration, were observed [70]. UCF spectrum shows an absorption bands at in 2919 cm^−1^, attributed to axial strain of group C-H [70], and 1604 cm^−1^ referring to C=C, characteristic of lignin [36], while on CF spectrum this band had decreasing. The band at 1736 cm^−1^ of the UCF spectrum refers to carbonyl groups (C=O) of the hemicellulose [71]. This band disappears in CF spectrum. Such results suggest the efficiency of the alkaline treatment of coconut fiber with regard to the disruption of the lignocellulosic material: break of the bind involving acetyl group of the hemicellulose and lignin, thus disintegrating the lignin components of the lignocellulosic material [72]. Therefore, it is suggested that the availability of the reactive hydroxyl groups in the CF was improved. Also, UCF and CF spectra showed absorption band at 3313 cm^−1^ corresponding to hydroxyl groups (OH) present on the surface of the support [36]. Characteristic bands of the support (1604 cm^−1^) and of the enzyme (1636 cm^−1^) can be visualized in CF-CB spectrum. Thus, the FTIR analysis suggests enzyme immobilization of the enzyme in CF.

CAMs spectra showed an absorption band at 1029 cm^−1^ assigned to C-O-C stretching vibration, characteristic of the alginate structure [73] (Figure 7). Also, CAMs and CAMs-CB spectra show absorption bands at 1410 cm^−1^ and 1595 cm^−1^ corresponding to COO^−^, referring to symmetrical stretching [42] and asymmetric stretching, respectively [74]. Absorption bands observed in the CAMs and CAMs-CB spectra in the 3000–3600 cm^−1^ range and at 2920 cm^−1^ are associated with the elongation of the O-H and vibration C-H, respectively [75]. CAMs-CB spectra show absorption band at 1636 cm^−1^ (amide I) indicating the enzyme presence in the immobilized system [59].

HB and HB-CB spectra showed vibration bands at 794 cm^−1^ (symmetric stretching) and 1066 cm^−1^ (asymmetric stretching) attributed to the siloxane groups (Si-O-Si) on the silica surface [76] HB-CB spectrum shows vibration band at 965 cm^−1^—referring to Si–OH [19]. HB-CB spectrum shows absorption band at 1636 cm^−1^ (amide I) indicating the enzyme presence in the immobilized system (Figure 7).

#### 2.5.3. Thermogravimetric Analysis (TGA)

Thermogravimetric analysis (TGA) is used to characterize materials through changes in physical and chemical properties such as dehydration and decomposition of the material due to the increase in temperature. Table 1 shows the mass loss (%) and moisture content (%) data by TGA of the samples: untreated coconut fiber (UCF), treated coconut fiber (TCF), calcium alginate microspheres (CAMs), SBA-15/albumin hybrid (HB); immobilized radish peroxidase by covalent binding (CB): HB-CB, CAMs-CB, CF-CB. 

The mass losses obtained by thermogravimetry were distinguished in three regions: region I (25–200 °C) corresponds to the mass losses related to dehydration and proteins; region II (200–600 °C) is linked to the decomposition of organic compounds, which in this study include alginate and coconut fiber supports, and silanol groups of the HB support; and the region III (above 600 °C) represents losses of mass resulting from final dehydroxylation reactions and carbonization of organic compounds. Thermal degradation of CF on region I is due to the water evaporation and volatile substances. In region II occurs cellulose degradation (290–360 °C). On the order hand, lignin presents higher thermal stability and degradation even above 500 °C [77]. Comparing the mass losses of UCF and CF, the higher values were observed for CF in the regions I and II (8.71% and 76.03%, respectively). However, CF exhibited less mass loss (11.39%) in the region III, this possibly due to delignification of the lignocellulosic material. The results suggest the presence of water and higher cellulose content due to the alkaline treatment. 

Analyzing the data set of each support, Table 1 shows an increase in mass losses in region I after the enzyme immobilization. In addition, the data of mass loss of biocatalysts immobilized by CB are higher than for IBs by PA. These results indicate a greater amount of water and protein molecules in IBs by CB. In addition, the results of mass loss in region I corroborate the percentage of moisture in the samples and the higher values of immobilization efficiency obtained by CB (CF-77.74 ± 0.42%), CAMs-11.83 ± 0.68%). For HB-CB the moisture content was the higher observed (16%), as well IY (89.99% ± 0.38). The results suggest immobilization of radish peroxidase by covalent binding is associated with greater conservation of the hydration layer on immobilized enzyme [48]. Consequently, an improvement in the catalytic activity of radish peroxidase was reached.

## 3. Materials and Methods

### 3.1. Preparation of the Supports

#### 3.1.1. Coconut Fiber (CF)

Green coconut was supplied by the Embrapa-Tabuleiros Costeiros (Aracaju-Brazil), processed in the knife mill and was subsequently dried at 70 °C obtaining the untreated coconut fiber (UCF). This material was pretreated according to Widnyana et al. [78] with modifications: 5 g of were transferred to Erlenmeyer flasks containing 250 mL of NaOH (5% (*w/v*)) and gently stirred at 28 °C for 24 h. Then, the material was washed with distilled water until for pH neutralization, dried in the oven at 90 °C/24 h and sieved for standard uniformity of t of 100 mesh. Treated coconut fiber was denominated as CF.

#### 3.1.2. Calcium Alginate Microspheres (CAMs)

Calcium alginate microspheres (CAMs) obtaining was performed following the procedure described in literature [79]. Two solutions were prepared: an aqueous solution of sodium alginate (5% (*w/v*)), and an aqueous solution of calcium chloride (1 M). Under magnetic stirring, the total sodium alginate solution was added drop by drop trough a glass burette (capacity of 100 mL) in the solution of calcium chloride. CAMs were filtered washed with distilled water to remove calcium chloride and dried at 40 °C for 12 h.

#### 3.1.3. SBA-15/Albumin Hybrid (HB)

The hybrid support was obtained using methodology for SBA-15 synthesis described in the literature with minor adaptations [47,80]. Bovine serum albumin 1% (*w/v*) (1.4 g) was dissolved in 30 mL of distilled water. To this solution, structure-directing agent Pluronic F127 (4 g) and 104 mL of HCl (2 M) were added. Then 8.0 mL of tetraethyl orthosilicate (TEOS) were added to the solution which was stirred at room temperature for 20 h. The mixture was poured into a reactor hermetically sealed and kept at 80 °C for 24 to 48 h under static conditions. The solid was recovered by filtration, washed with distilled water for pH neutralization and dried at room temperature by 24 h. The surfactant agent and albumin were removed by calcination at 550 °C/6 h—heating rate of 1.8 °C·min^−1^. Finally, the particles were reduced to 32 mesh.

### 3.2. Preparation of the Radish Crude Extract: Alternative Source of Peroxidase 

Radish samples were obtained from local market in Aracaju-SE—Brazil. The extraction of the crude enzyme was performed according methodology established in the literature [17]. In summary, the radish root was washed, peeled and stored in 250 g fractions at −10 °C. A sample of the vegetable was homogenized with 100 mL of 100 mM phosphate buffer (pH 6.5), filtered and centrifuged at 6500 rpm for 30 min at 4 °C. Protein concentration was determined by Bradford method using bovine serum albumin as standard [81].

### 3.3. Enzyme Immobilization

Radish peroxidase was immobilized by physical adsorption (PA) and covalent binding (CB) according to Queiroz et al. [35] with minor modifications. Protein loading effect was evaluated (1.3–6.5 mg protein/g support). The immobilizations on the CF and HB supports were carried out in sodium phosphate buffer (Na_2_HPO_4_·2H_2_O) 100 mmol L^−1^ pH 8.0. Immobilization processes on CAMs were conducted in hexane for mechanical preservation of the material. 

For immobilization by covalent binding the CF, CAMs and HB supports (1 g) were previously silanized with (3-aminopropyl) triethoxysilane (APTES) 0.5% (*v/v*) and posteriorly activated with 4.6 mL of glutaraldehyde 2.5% (*v/v*) for 1 h at room temperature. The activated supports were washed with 3 portions of 10 mL of distilled water and were vacuum filtered. Non-activated CF and CAM supports were used for enzyme immobilization by PA technique.

For both immobilization process (CB and PA), 1 g of the support previously dried was suspended in 5.0 mL of the respective immobilization medium and keeping under mechanical agitation for 15 min. After, required amount of protein of the crude radish extract according to loading evaluation was added and final volume completed to 15 mL with immobilization medium. The mixture remained under stirring for 3 h at 25 °C, subsequently was stored at 4 °C under static condition for 24 h. Then, the immobilized biocatalysts (IBs) were washed and filtered to remove the non-immobilized enzymes. For the immobilization processes on CF and HB the filtrates were reserved for the measurement of peroxidase activity. 

### 3.4. Peroxidase Activity Assay

Tetraguaicol formation (product of guaiacol oxidation) was monitored spectrophotometrically at 470 nm by UV-Vis spectrophotometer (Biochrom Libra S22, Biochrom, Cambridge, United Kingdom) for three minutes (ε_tetraguaiacol_: 26.6 mM^−1^·cm^−1^) to determine peroxidase activity, according to literature [4]. Reaction medium containing: 2.76 mL of phosphate buffer 100 mM (pH 6.0); 0.04 mL of crude radish extract or filtrates of the immobilization processes; 100 μL of guaiacol solution 100 mM and 100 μL of hydrogen peroxide (H_2_O_2_) 2.0 mM at 25 °C. For immobilized biocatalysts on CAMs were used 50 mg of IB. An enzyme unit (U) was defined as the amount of enzyme capable of providing 1 μmol of product in 1 min at 25 °C in pH 6.0.

The efficiencies of immobilization of radish peroxidase were calculated in two forms: activity recovery (AR) for IB on CAMs—Equation (1); immobilization yield (IY%) for IBs on CF and HB—Equation (2).

(1) $$AR\%=\frac{\left(Immobilized\;activity\right)\;}{\left(peroxidase\;activity\;offered\right)}\times 100$$

(2) $$IY\%=\frac{\left(peroxidase\;activity\;offered-peroxidase\;remaining\;in\;the\;filtrate\right)}{\left(peroxidase\;activity\;offered\right)}\times 100$$

### 3.5. Characterization of the Immobilized Biocatalysts (IBs)

#### 3.5.1. Effect of pH, Temperature and Operational Stability

The biochemical characterization of the free enzyme and IBs were performed in order to determine optimum pH/ temperature, according to Al-Sa’ady et al. [18], with minor adaptations. For pH study, the peroxidase activity was evaluated in buffer solutions 0.1 M at different pHs: sodium acetate (pH 3–5), sodium phosphate (pH 6–8, 0.1 M) and sodium hydroxide (pH 9). For optimum temperature determination, the peroxidase activity of the crude radish extract and immobilized enzymes was measured between 25–60 °C. The results were expressed in terms of relative activity, using highest activity value obtained as reference (100%).

Operational stability of the IBs was performed according to item 3.4 by guaiacol oxidation in consecutive batches using 30 mg of IBs. At the end of each cycle the IB was previously washed. The results were expressed in terms of residual activity (RA) considering RA of 100% for initial cycle. Cycles were repeated until the activity reached less than 50% of the initial activity.

#### 3.5.2. Scanning Electron Microscopy (SEM), Fourier-Transform Infrared Spectroscopy (FTIR), Thermogravimetric Analysis (TGA) 

Scanning electron microscopy was used to determine the morphological features of the supports and immobilized biocatalysts. The samples were previously metalized using the Spitter Coater EMITECH Model: K450. For Scanning Electron Microscope with X-ray Dispersive Energy Detector, model Leo 440i (SEM), brand: LEO Electron Microscopy/Oxford, was used. Magnifications of 500× for CF, and 100× for CAMs and HB were applied.

Fourier-transform infrared spectroscopy (FTIR) analyses were performed using AGILENT CARY 630 FTIR spectrometer (Agilent Technologies, Santa Clara, CA, USA) equipped with a diamond/zinc selenide crystal (ZnSe) and ATR (Attenuated Total Reflection) device, spectral scale 4000–600 cm^−1^ and resolution of <2 cm^−1^ and processed for automatic data acquisition by Agilent MicroLab PC software.

Thermogravimetric analyses were performed using 5.0 mg of samples under argon atmosphere in the range of 25 to 900 °C using heating rate of 10 °C/min on a TA-Q50 equipment. TGA curves were analyzed using the TA universal analysis software.

### 3.6. Statistical Analysis

All the experiments were realized in triplicate and the experimental results were expressed as standard error of the mean (mean ± S.E) and represented as error bars in figures. Data were analyzed for statistical significance by analysis of variance (ANOVA) followed by Tukey post-test (*p* < 0.05) (Prims GraphPAD^®^ 7.0). 

## 4. Conclusions

Crude radish peroxidase, a low-cost enzyme, was successfully immobilized on CF and HB supports. Immobilization processes resulted in improvement or maintenance of the original enzyme’s biochemical characteristics. Covalent binding (CB) technique was the most suitable technique. SBA15/albumin hybrid (HB) support provided higher immobilization recovery (89.99% ± 0.38) and allowed the reuse of the immobilized biocatalyst in four cycles, making HB-CB promising for future applications.

## Figures and Tables

**Table 1:** Mass loss data (%) and moisture content (%) by TGA. Untreated coconut fiber (CF), treated coconut fiber (CF), calcium alginate microspheres (CAMs), SBA-15/albumin hybrid (HB). Immobilized radish peroxidase by covalent binding (CB): HB-CB, CAMs-CB, CF-CB.

Mass Loss (%)				Moisture Content ^1^ (%)
Sample	Region I 25–200 °C	Region II 200–600 °C	Region III Above 600 °C	
UCF	7.18	65.36	12.37	----
CF	8.71	76.03	11.39	3.80 ± 0.37
CF-PA	10.28	72.17	13.31	5.40 ± 0.05
CF-CB	11.40	73.97	11.73	3.78 ±0.22
CAM	13.99	43.86	14.77	1.46 ± 0.14
CAM-PA	22.24	44.64	20.40	1.56 ± 0.25
CAM-CB	35.80	43.20	15.12	1.47 ± 0.03
HB	9.31	3.91	1.21	10.99 ± 0.64
HB-CB	17.77	4.00	0.03	16.07 ± 0.29

^1^ Determined by Karl Fisher method.
